# DDML: Multi-Student Knowledge Distillation for Hate Speech

**DOI:** 10.3390/e27040417

**Published:** 2025-04-11

**Authors:** Ze Liu, Zerui Shao, Haizhou Wang, Beibei Li

**Affiliations:** School of Cyber Science and Engineering, Sichuan University, Chengdu 610211, China; liuze@stu.scu.edu.cn (Z.L.); whzh.nc@scu.edu.cn (H.W.); libeibei@scu.edu.cn (B.L.)

**Keywords:** DDML, knowledge distillation, hate speech detection

## Abstract

Recent studies have shown that hate speech on social media negatively impacts users’ mental health and is a contributing factor to suicide attempts. On a broader scale, online hate speech can undermine social stability. With the continuous growth of the internet, the prevalence of online hate speech is rising, making its detection an urgent issue. Recent advances in natural language processing, particularly with transformer-based models, have shown significant promise in hate speech detection. However, these models come with a large number of parameters, leading to high computational requirements and making them difficult to deploy on personal computers. To address these challenges, knowledge distillation offers a solution by training smaller student networks using larger teacher networks. Recognizing that learning also occurs through peer interactions, we propose a knowledge distillation method called Deep Distill–Mutual Learning (DDML). DDML employs one teacher network and two or more student networks. While the student networks benefit from the teacher’s knowledge, they also engage in mutual learning with each other. We trained numerous deep neural networks for hate speech detection based on DDML and demonstrated that these networks perform well across various datasets. We tested our method across ten languages and nine datasets. The results demonstrate that DDML enhances the performance of deep neural networks, achieving an average F1 score increase of 4.87% over the baseline.

## 1. Introduction

Hate speech on social media can have adverse effects on users. Surveys indicate that online hate speech contributes to suicide attempts and suicidal ideation, as well as mental health issues [1]. On a macro level, online hate speech can exacerbate social polarization and undermine social stability [2]. As social media continues to evolve and its content expands, the proliferation of hate speech also increases [3], making automatic detection of hate speech a crucial element in combating it. Globally, a range of laws and policies have been introduced to maintain a clear online legal environment for citizens. These regulations not only standardize the content of online speech content, but also set higher standards for online platform operators. The severity of the situation has prompted social media platforms and academic researchers to propose traditional machine learning and deep learning solutions for automatic detection [4] and early detection [5] of online hate speech. Among these solutions, large pretrained language models (LLMs) have demonstrated their superiority in many natural language processing (NLP) tasks [6] and have also shown excellent performance in hate speech detection [7].

However, existing research has primarily focused on detecting hate speech in English and Western cultural contexts, neglecting a significant amount of online hate speech from other languages and cultures. This limits the global identification and control of aggressive language. There has been very little research on multilingual hate speech detection. One feasible method is to fine-tune pretrained language models [8,9]. At the same time, deep neural networks used in hate speech detection often have considerable depth or breadth, and typically contain numerous parameters. This results in high computational requirements, restricting the application of these models on personal computers.

Knowledge distillation is a model compression technique that enhances the performance of a smaller model (the student) by transferring knowledge from a larger, high-performing model (the teacher). This method allows the student model to learn and mimic the teacher model’s behavior while maintaining a smaller model size and lower computational cost. Knowledge distillation is an excellent solution for addressing the deployment challenges of large models.

In real-life scenarios, students in a class learn not only from the teacher but also from their peers, which reinforces the knowledge gained from the teacher. Similarly, we believe that the student network can obtain knowledge not only from the teacher network but also from other student networks. In this paper, we aim to address the issues mentioned above by using a deep neural network with fewer parameters. To achieve excellent detection accuracy with a smaller deep neural network, we propose a method similar to model distillation [10], which we call Deep Distill–Mutual Learning (DDML).

Model distillation typically starts with a larger and more powerful pretrained teacher network which transfers knowledge to a smaller untrained student network. Our proposed DDML requires a powerful teacher network along with two or more student networks. While the teacher network transfers knowledge to the student networks in one direction, the student networks also learn from each other to solve the problem. Specifically, each student network uses three types of loss: the traditional supervised learning loss, the loss of the teacher network’s output, and the imitation loss, which mimics other students. Our experiments show that the performance of student networks trained in this way surpasses that of student networks trained using the traditional model distillation method.

It may not be immediately clear why the proposed DDML learning strategy outperforms the traditional distillation method. In this regard, intuition can be gained from the following. Each student network is trained using both the true labels and the outputs of the powerful teacher model, which generally improves the performance of the student networks. However, because each student network has a different architecture and initial conditions, they learn different representations, leading to varying predictions. These differences provide additional knowledge during the training process, helping the student networks to achieve better performance.

The main contributions of this paper are as follows:

1. First, we develop a knowledge distillation-based deep neural network training method called DDML for online hate speech detection. This method facilitates the construction of a lightweight hate speech detection model, making it well suited for deployment on resource-constrained devices.

2. Second, we leverage various deep neural networks, including BERT [11] and XLM-R [12], and train them using the proposed DDML method. On the one hand, this approach ensures high detection accuracy across different architectures, while on the other it enables robust performance in monolingual, multilingual, and machine translation-based hate speech detection tasks.

3. Third, we conduct extensive experiments across ten languages and nine datasets to validate the effectiveness of the proposed method. The results demonstrate that our approach achieves an average F1 score improvement of 4.87% over the baseline, highlighting its superior performance in diverse linguistic settings.

## 2. Related Works

In this section, we introduce previous work related to hate speech detection and knowledge distillation.

### 2.1. Hate-Speech Detection

In recent years, hate speech detection has gained significant attention. Historically, research has predominantly focused on English due to the scarcity of datasets, with some studies extending to monolingual hate detection in other languages such as German [13], Greek [14], and others. However, hate speech is a global issue and is not confined to a single language. The availability of more diverse resources is crucial for advancing automatic detection tools. Significant strides have been made in providing multilingual resources; for instance, SemEval offers a series of high-quality multilingual labeled datasets [15], while EVALITA provides a shared framework for consistently evaluating various systems and methods [16]. Additionally, Facebook’s transformer-based XLM-R model supports over one hundred languages through pretraining [12]. Prior studies on multilingual hate speech detection have explored several approaches, including both transfer learning, which leverages large pretrained models and shared word embeddings across multiple languages to enhance detection capabilities, and zero-shot learning, which aims to detect hate speech in languages for which no labeled data are available.

#### 2.1.1. Transfer Learning for Hate Speech Detection

The core idea of transfer learning involves initially training a network extensively on a high-resource language such as English in order to learn general features. This network is then used as a starting point and fine-tuned with a small amount of data in a new language. Ranasinghe et al. [7] were the first to apply cross-lingual contextual word embeddings in offensive language identification, projecting predictions from English to other languages using benchmarked datasets from shared tasks on Bengali, Hindi, and Spanish. Aluru et al. [8] conducted the first extensive evaluation of multilingual hate speech detection using a dataset covering nine languages from sixteen different sources. They found that LASER [17] embeddings with logistic regression performed the best in low-resource scenarios, while in high-resource scenarios BERT-based models [11] significantly outperformed other methods. Previous research has also explored translation-based solutions to address the shortage of hate speech data in low-resource languages. These approaches depend heavily on the quality of translation APIs, and as such can incur significant overhead due to the large number of translation requirements. Additionally, during the fine-tuning process in transfer learning, models may experience “catastrophic forgetting” of knowledge acquired during pretraining, particularly when the target tasks differ significantly from the source tasks [18].

#### 2.1.2. Zero-Shot Learning for Hate Speech Detection

Zero-shot learning involves training models to recognize unseen classes by learning intermediate attribute classifiers or a mixture of seen class proportions. This approach enables the network to generalize to new languages without additional training data. Endang et al. [19] explored hate speech detection in low-resource languages by first transferring knowledge from English in a zero-shot learning manner. They proposed two joint learning models, and achieved state-of-the-art performance in most languages. Jiang et al. [20] developed a tailored architecture based on frozen pretrained transformers for cross-lingual zero-shot and few-shot learning using the HatEval dataset. Their approach achieved competitive results on the English and Spanish subsets. Despite these advancements, zero-shot learning for hate speech detection has notable limitations. Sahin et al. [21] assessed the zero-shot performance of various models trained on different hate speech datasets. Their results indicated that transformer-based language models outperformed traditional models, with few-shot methods surpassing zero-shot methods. However, Nozza et al. [22] found that zero-shot learning methods performed poorly in detecting hate speech against women. In their research, they argued that hate speech detection is language-specific that and natural language processing methods must account for this specificity in order to be effective. Similarly, Montariol et al. [23] highlighted the limitations of hate speech models in rigorous experimental settings across various domains and languages.

### 2.2. Knowledge Distillation

In recent years, deep neural networks have achieved remarkable success in both industry and academia [24]. This success is largely due to deep learning’s ability to scale with large datasets and manage billions of model parameters. However, deploying these large models on resource-constrained devices such as embedded systems presents significant challenges. These challenges arise not only from these models’ high computational complexity but also from their substantial storage requirements. To address these challenges, various model compression and acceleration techniques have been developed. Among these techniques, knowledge distillation has garnered significant attention for its effectiveness in training smaller student models from larger teacher models. In knowledge distillation, the type of knowledge being transferred plays a crucial role in the student models’ performance. This knowledge is generally categorized into three types: (1) response-based knowledge, (2) feature-based knowledge, and (3) relationship-based knowledge.

#### 2.2.1. Feature-Based Knowledge

Feature-based knowledge distillation leverages intermediate-layer feature activations from the teacher model, which are then used to train the student model. The goal is for the student model to learn similar feature representations as the teacher model. Romero et al. [25] extended knowledge distillation by allowing the training of student models that are deeper than the teacher model. They used intermediate representations learned by the teacher as hints to enhance the student model’s training process and final performance. Ding et al. [26] demonstrated that the performance of student convolutional neural networks (CNNs) can be significantly improved by accurately defining CNN attention and by having the student CNN mimic the attention map of the more powerful teacher network. Chen et al. [27] introduced Semantic Calibration for Cross-Layer Knowledge Distillation (SemCKD), which uses an attention mechanism to automatically assign the appropriate target layer of the teacher model to each layer of the student model. However, knowledge-based distillation might not fully capture global structural information, which can result in the student model failing to learn all the useful features of the teacher model [28].

#### 2.2.2. Relation-Based Knowledge

Relation-based knowledge distillation focuses on capturing and transferring the relationships between feature maps in a neural network, such as correlations, graph representations, and similarity matrices. This approach aims to convey these structural relationships from the teacher model to the student model. Park et al. [29] introduced Relational Knowledge Distillation (RKD), which transfers mutual relationships between data examples. They proposed using distance and angle distillation losses to penalize structural differences in these relationships. Experiments across various tasks demonstrated that RKD significantly improves the performance of the student model. To leverage knowledge from multiple teacher models, Zhang and Peng [30] created two graphs, one of which uses the logits of each teacher model as nodes while the other uses the features. Through these graphs, their approach models the importance and relationships of different teachers prior to transferring knowledge. Lee et al. [31] developed a method for distilling dataset-based knowledge from a teacher model using an attention network. Their approach involves embedding the teacher model’s knowledge into a graph via multi-head attention (MHA) and performing multitask learning to provide the student model with a relational inductive bias. However, relation-based distillation can be complex and computationally intensive, as it requires capturing and utilizing structured relationships between the outputs of the teacher model [32].

#### 2.2.3. Response-Based Knowledge

Our proposed DDML proposed incorporates response-based distillation as its initial step. Response-based knowledge distillation focuses on the final output layer of the teacher model, aiming to train the student model to replicate the teacher model’s prediction results. This is typically achieved by minimizing the difference between the logits of the student and teacher models, using soft targets as the source of knowledge. Hinton et al. [10] introduced a distillation method based on soft targets, where the soft targets represent the predicted probability distribution of the teacher model for each class. This approach allows the student model to learn the teacher model’s probability distribution, thereby enhancing the student model’s generalization ability. Additionally, Kim et al. [33] proposed a class distance loss that helps the teacher network to create a densely clustered vector space. This facilitates easier learning for the student network by making the structure of the teacher’s feature space more accessible.

## 3. Methodology

In this section, we illustrate the overall system architecture of DDML using two student networks as examples, discuss the calculation of the DDML loss function, describe the optimization process of DDML, and finally extend DDML to more than two student networks.

### 3.1. System Overview

**Figure 1:**
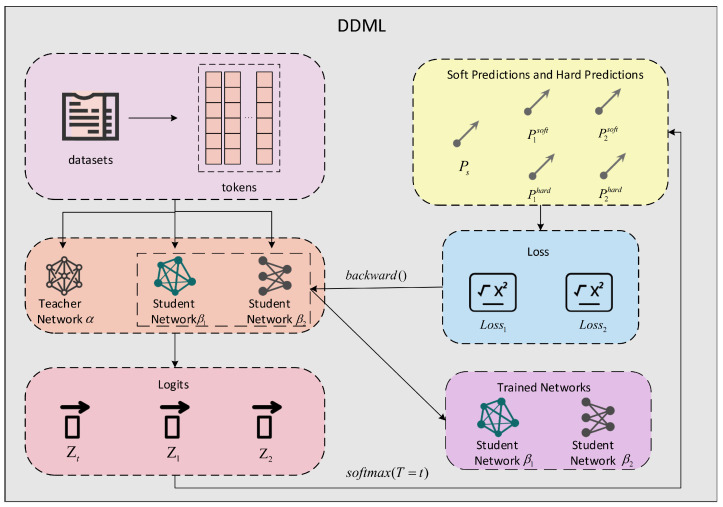
DDML schematic with two student networks. In DDML, the teacher network does not perform backpropagation, which means that the parameters of the teacher network are not updated, while for the student network the opposite holds. It should be noted that the two student networks need to either use different types of models or use models with different pretraining levels.

In Section 3.1 and Section 3.2, we illustrate the use of two student networks as an example. The overall framework of DDML with two student networks is depicted in Figure 1.

Given a sentences containing *N* words $X={\left\{x_{i}\right\}}_{i=1}^{N}$ from *M* classes, we denote the corresponding label set as $Y={\left\{y_{i}\right\}}_{i=1}^{M}$ with $y_{i}\in \left\{1,2,\ldots ,M\right\}$. We first segment the sentence *X* to obtain its corresponding tokens $E={\left\{e_{i}\right\}}_{i=1}^{N}$, then input the tokens into the teacher network α, student network $\beta_{1}$, and student network $\beta_{2}$ and obtain the output logits *Z* of the three networks:

(1) $$\left\{\begin{array}{c} Z=\{Z_{t},Z_{1},Z_{2}\},\\ Z_{t}=F_{t}\left(E\right),\\ Z_{1}=F_{1}\left(E\right),\\ Z_{2}=F_{2}\left(E\right). \end{array}\right.$$

Here, $Z_{t}={\left\{z_{t}^{i}\right\}}_{i=1}^{M}$, $Z_{1}={\left\{z_{1}^{i}\right\}}_{i=1}^{M}$, and $Z_{2}={\left\{z_{2}^{i}\right\}}_{i=1}^{M}$; $Z_{t}$, $Z_{1}$, and $Z_{2}$ are the logits output by networks α, $\beta_{1}$, and $\beta_{2}$ respectively; and $F_{t}$, $F_{1}$, and $F_{2}$ represent the operation process of networks α, $\beta_{1}$m and $\beta_{2}$, respectively. The probabilities of class m for sentence *X* are computed as follows:

(2) $$P_{t,m}\left(X,T\right)=\frac{exp(z_{t}^{m}/T)}{\sum_{m=1}^{M}exp(z_{t}^{m}/T)},$$

(3) $$P_{i,m}^{j}\left(X,T\right)=\frac{exp(z_{i}^{m}/T)}{\sum_{m=1}^{M}exp(z_{i}^{m}/T)},$$

where i∈{1,2}, {j,T}∈{soft,1},{hard,t(t≠1)}, the parameter *T* is the distillation temperature, $P_{t,m}\left(X,T\right)$ is the probability of the teacher network α classifying sentence *X* and finding that it belongs to category *m*, and $P_{i,m}^{j}\left(X,T\right)$ is the probability of the student network $\beta_{i}$ classifying sentence *X* and finding that it belongs to category *m*. Soft labels and hard labels are obtained by the following formula:

(4) $$T_{s}=max\left({\left\{P_{t}^{m}\right\}}_{m=1}^{M}\right),$$

(5) $$S_{i}^{j}=max\left({\left\{P_{i}^{m}\right\}}_{m=1}^{M}\right),$$

where i∈{1,2} and j∈{soft,hard}. When T=1, the hard predictions $S_{1}^{hard}$, $S_{2}^{hard}$ of the student network $\beta_{1}$, $\beta_{2}$ can be obtained by Formula (5), while when T=t and t≠1, the same formula can be used to obtain the soft predictions $S_{1}^{soft}$, $S_{2}^{soft}$ of the student network $\beta_{1}$, $\beta_{2}$, where $T_{s}$ represents the soft predictions obtained by teacher network α.

### 3.2. Loss Function of DDML

**Figure 2:**
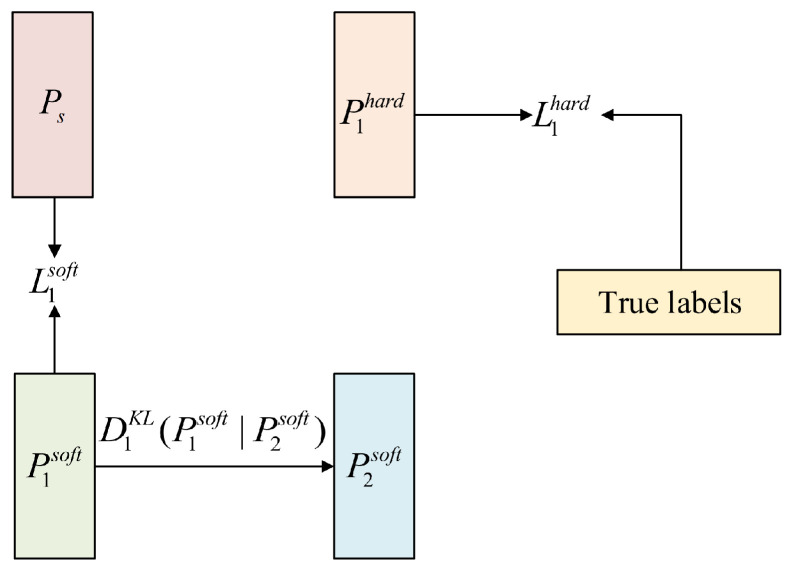
The loss of the student network $\beta_{1}$ consists of three parts: $L_{1}^{soft}$, $L_{1}^{hard}$, and $D_{KL}\left(P_{1}^{soft}|P_{2}^{soft}\right)$.

**Figure 3:**
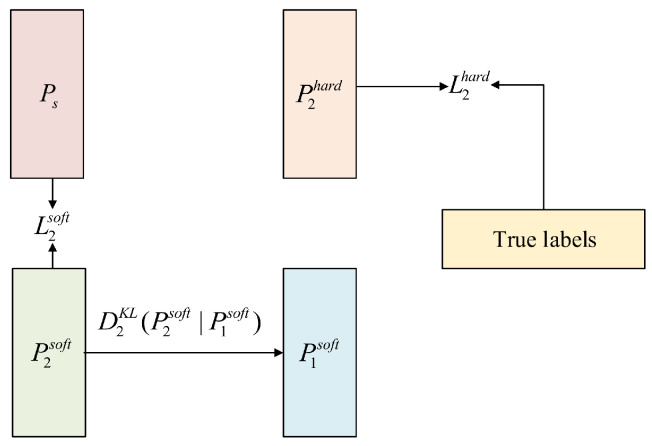
The loss of the student network $\beta_{2}$ consists of three parts: $L_{2}^{soft}$, $L_{2}^{hard}$, and $D_{KL}\left(P_{2}^{soft}|P_{1}^{soft}\right)$.

For the loss function, we choose the cross-entropy loss. In order to calculate the difference between the prediction vectors $P_{1}$, $P_{2}$ of the two student networks, we also calculate the Kullback–Leibler divergence (KL divergence) between $P_{1}$ and $P_{2}$. The method for calculating the loss function of the two student networks $\beta_{1}$ and $\beta_{2}$ is shown in Figure 2 and Figure 3.

For student network $\beta_{1}$, three losses are used in its loss function: $L_{1}^{soft}$, $L_{1}^{hard}$, and $D_{KL}\left(P_{1}^{soft}|P_{2}^{soft}\right)$. The calculation of these three losses is as follows:

(6) $$L_{1}^{hard}=-\sum_{i=1}^{N}\sum_{m=1}^{M}I\left(y_{i},m\right)\log\left(P_{1}\right),$$

(7) $$\begin{array}{cc} L_{1}^{soft}= & -\sum_{i=1}^{N}\sum_{m=1}^{M}I\left(P_{t}^{m},P_{1}^{m}\right)\log\left(P_{1}\right), \end{array}$$

(8) $$\begin{array}{c} D_{KL}\left(P_{1}^{soft}|P_{2}^{soft}\right)=\sum_{n=1}^{N}\sum_{m=1}^{M}P_{1}^{soft}log\frac{P_{1}^{soft}}{P_{2}^{soft}}, \end{array}$$

where

(9) $$I\left(x,y\right)=\left\{\begin{array}{cc} 1, & \mathrm{if}\;x=y\\ 0, & \mathrm{if}\;x\neq y. \end{array}\right.$$

The method for calculating the final loss $L_{1}$ of student network $\beta_{1}$ is as follows:

(10) $$L_{1}=a\cdot L_{1}^{hard}+b\cdot L_{1}^{soft}+c\cdot D_{KL}\left(P_{1}^{soft}|P_{2}^{soft}\right).$$

### 3.3. Optimization of DDML

The key difference between traditional model distillation and DDML is the latter’s incorporation of a mutual learning strategy. In DDML, multiple student networks are optimized simultaneously, and learn not only from the teacher network but also from each other. This mutual learning approach is embedded throughout the entire training process. For instance, with two student networks, the predictions of each network are computed, then the Kullback–Leibler (KL) divergence between the predictions of one network and those of the other is incorporated into the loss function.

### 3.4. Extension to Multiple Student Networks

Assuming that there are *K* student networks, the loss function calculation of the student network *i* becomes

(11) $$\begin{array}{cc} L_{i}= & \;a\cdot L_{i}^{hard}+b\cdot L_{i}^{soft}+\frac{c}{K-1}\cdot \sum_{k=1,k\neq i}^{N}D_{KL}\left(P_{i}^{soft}|P_{k}^{soft}\right). \end{array}$$

Equation (11) shows that in the case of *K* networks, each student takes the teacher network and the other K−1 student networks in DDML as its teacher, that is, its imitation target. It is worth noting that Equation (10) is a special case of Equation (11) in which K=2. It should be noted that we have added the coefficient $\frac{1}{K-1}$ to ensure that the training is mainly directed by supervised learning of the true labels and teacher network’s soft predictions. The algorithm is summarized in Algorithm 1.
**Algorithm 1** Deep Distill–Mutual Learning1:**Initialize:** Initialize *N* student networks ${\left\{\beta_{i}\right\}}_{i=1}^{N}$ to different conditions;2:**Input:** training set *X* and labels *Y*;3:**repeat**:4:    Sample *x* from training set *X* randomly;5:    Compute predictions and losses;6:    Compute stochastic gradient and update student network $\beta_{i}\left(i\in 0,1,\ldots ,N\right)$:(12)$$\beta_{i}\leftarrow \beta_{i}+\gamma_{t}\frac{\delta L_{i}}{\delta \beta_{i}};$$7:    Update predictions;8:    (13)epoch←epoch+19:**until** :(14)epoch==epoches

## 4. Experiment

In the following, we describe the use of the design from Part III to conduct experiments. First, we introduce the datasets, baseline, transformer-based models, and implementation details, then the use of the acquired data to conduct experiments and collect results.

We implemented all networks and training procedures in PyTorch (version 2.0.1+cu117) and conducted all experiments on an NVIDIA RTX A6000 GPU (made by NVIDIA, in Santa Clara, CA, USA). For the transformer-based models, we used the HuggingFace transformer library and initialized the selected pretrained models with weights provided by this library.

### 4.1. Datasets

To detect multilingual hate speech, we conducted experiments on the following nine datasets:

SemEval2020 [15], which contains four low-resource languages: Arabic, Danish, Swiss, and Turkish.

HASOC2020 [34], which contains three languages: English, German, and Hindi.

GermEval 2018 [35]: Haspeede2 [36], HaterNET [37], OLID [38], and COLD [39] are single-language datasets respectively consisting of German, Italian, Spanish, English, and Chinese.

OffComBr-2 [40] is a dataset of hate speech comments in Portuguese from the Brazilian news website (accessed on 19 November 2024) https://g1.globo.com/.

Twitter Racism [22], the zero-shot learning dataset proposed by Nozza, is insufficient for detection of hate speech against women. Thus, to verify the performance of the network trained by DDML in the detection of such hate speech, we selected the Twitter Racism Dataset from HuggingFace, which is an English dataset of hate speech against women.

To verify the performance of the network trained by DDML on other types of hate speech, we also selected the public dataset SexismDetection from HuggingFace, which is an English hate speech dataset focusing on sexism. Table 1 provides the basic statistics of these monolingual datasets after merging the training and the test sets.

To verify the performance of the student networks on a large multilingual dataset, we combined the eight datasets used by Awal et al. [41] in their HateMAML study: English (OLID), Arabic (SemEval), Danish (SemEval), Turkish (SemEval), Greek (SemEval), German (HASOC), Italian-news (Haspeede), and Italian-tweets (Haspeede). The result was a new large-scale dataset named UniHate.

To create a larger dataset while maintaining consistency with the work of Firmino et al. [42], we combined the Haspeede [36], OffCombr-2 [40], and HaterNet [37] datasets, shuffled their order, and synthesized a new multilingual dataset named Multilingual. This multilingual dataset was then translated into English using Google Translator and DeepL Translator. Both the merged multilingual dataset and the translated dataset were used in our experiments (see Table 2).

### 4.2. Baseline

DDML is a network training method for hate speech detection. Therefore, we used the following methods as baselines for the experiment.

HateMAML [41]: Awal et al. proposed the HateMAML method for hate speech detection in 2024. They conducted experiments on OLID [38] (HatEval2019), SemEval2020 [15], HaSpeedDe [36], and HASOC2020 [34] datasets, achieving good results. Therefore, we used HateMAML as one of the baselines for these datasets.

Cross-Lingual Learning for Hate Speech Detection (CLHSD) [42]: Firmino et al. conducted experiments on datasets consisting of Italian (Haspeede [36]) and Portuguese (OffComBr-2 [40]) using various training strategies, including zero-shot transfer (ZST) learning, joint learning (JL), and cascade learning (CL), demonstrating the effectiveness of cross-lingual learning in hate speech detection.

Transfer Learning For Hate Speech Detection (TLHSD) [43]: Fillies et al. used transfer learning methods to classify hate speech in German, Italian, and Spanish on the GermEval 2018 [35], Haspeede [36], and HaterNET [37] datasets in 2023, achieving good results. Therefore, we used the transfer learning method as one of the baselines for the above three datasets.

### 4.3. Transformer-Based Models

This experiment utilized different versions of the BERT [11] network and various versions of the XLM-R [12] network. The different BERT versions included mBERT (bert-base-multilingual-uncased) [11], distillBERT (distillbert-base-uncased) [44], GermanBERT (bert-base-german-cased), ItalianBERT (bert-base-italiana-cased), BETO (bert-base-spanish-wwm-cased) [45], and BERTimbau (bert-base-portuguese-cased). The different XLM-R versions included XLM-R (xlm-roberta-large) [12] and XLM-R (xlm-roberta-base) [12]. For training neural networks based on DDML, XLM-R (xlm-roberta-large) [12] was uniformly selected as the teacher network. The specific networks and their parameter sizes are shown in Table 3.

The different versions of the BERT network are all based on the transformer encoder model, which focuses on understanding the context of text data. These networks use a multilayer stacked transformer encoder architecture in which all layers process the input in parallel while leveraging bidirectional context to learn semantic representations. At the same time, all versions of the BERT network are bidirectional encoders that are pretrained through two tasks, namely, masked language model (MLM) and next-sentence prediction (NSP).

As with BERT, The XLM-R series of models is also based on the transformer encoder architecture. XLM-R is a multilingual version of BERT designed to achieve cross-lingual transfer learning via pretraining on multiple languages. The pretraining approach of XLM-R is similar to that of BERT, using the masked language model (MLM) for training; the difference lies in its use of a large amount of multilingual data (such as datasets from 100 languages). XLM-R achieves cross-lingual generalization by training on these languages in a unified manner.

For the transformer-based models, we used the HuggingFace transformers library and initialized the selected pretrained models with the weights provided by this library.

## 5. Results and Analysis

We utilized four evaluation metrics: accuracy, precision, recall, and F1 Score (macro). The following descriptions use the abbreviations TP (true positive), TN (true negative), FP (false positive), and FN (false negative). Accuracy is the ratio of the number of samples predicted correctly by the model to the total number of samples:

(15) $$\begin{array}{c} Accuracy=\frac{TP+TN}{TP+TN+FP+FN}. \end{array}$$

Precision refers to the proportion of true positive samples among all samples predicted as positive by the model:

(16) $$\begin{array}{c} Precision=\frac{TP}{TP+FP}. \end{array}$$

Recall refers to the proportion of true positive samples that are successfully identified by the model out of all actual positive samples:

(17) $$\begin{array}{c} Recall=\frac{TP}{TP+FN}. \end{array}$$

Finally, the F1 score is particularly useful in cases where the dataset is imbalanced, i.e., when the number of positive and negative samples differs greatly:

(18) $$\begin{array}{c} F1\;Score=\frac{2\cdot TP}{2\cdot TP+FP+FN}. \end{array}$$

Precision and recall often constrain each other; increasing precision (i.e., reducing false positives) may lead to a decrease in recall (more false negatives), while increasing recall (reducing false negatives) may cause the precision to drop (more false positives). The F1 score is the harmonic mean of the precision and recall, and as such can be used as a comprehensive metric to evaluate a model.

Our experiment was conducted five times fir each group. Therefore, in the tables in this section, specifically Table 4, Table 5, Table 6, Table 7 and Table 8, each $x_{y}$ represents *x* as the average value of the F1 score or accuracy, with *y* as the standard deviation.

### 5.1. Comparison Experiment Between DDML and HateMAML

HateMAML was proposed by Awal et al. [41], who conducted experiments on the OLID, SemEval, HASOC, and Haspeede datasets using mBERT and XLM-R (xlm-roberta-base) networks. Therefore, our experimental design for these datasets used the same two networks as student networks. Furthermore, we incorporated the large multilingual UniHate dataset alongside these datasets for comparison.

Table 4 presents the accuracy, precision, recall, and F1 score results of the XLM-R and mBERT student networks trained using DDML on the OLID, SemEval2020, HaSpeedDe, and HASOC2020 datasets in comparison with those of HateMAML.

From the overall experimental data, it is evident that our DDML method not only outperforms HateMAML in terms of accuracy but also achieves generally better results in precision, recall, and F1 score. This advantage is validated across different models (mBERT and XLM-R) and multiple language datasets, demonstrating that DDML has stronger generalization ability and robustness compared to HateMAML.

The experimental data show that DDML demonstrates significant improvements across multiple language datasets, proving its applicability to languages beyond English such as Arabic, Danish, Turkish, and Italian. Even for high-resource languages such as English and German, DDML still outperforms HateMAML, suggesting that it can further optimize models and enhance classification performance even when large amounts of data are available. For example, on the German (HASOC) dataset, the F1 score of mBERT trained with DDML reached 0.8125, whereas HateMAML achieved only 0.7772, demonstrating that DDML consistently provides performance gains. For low-resource languages such as Arabic and Turkish, the improvements with DDML are even more pronounced, with the largest performance boost observed on the Arabic dataset. Specifically, the F1 score of XLM-R trained with DDML was 33% higher than that of HateMAML. At the same time, the network trained with DDML shows a relatively balanced performance in both precision and recall on most datasets, indicating that the network handles positive and negative samples in a balanced manner without a significant bias toward any particular class. This suggests that DDML is particularly well suited for tasks with limited data and complex distributions, as it can make more effective use of the available samples to enhance model generalization. Notably, DDML performed well on the large-scale multilingual UniHate dataset, surpassing HateMAML in all four evaluation metrics. This further demonstrates that networks trained with DDML have stronger generalization capabilities on large datasets and a better understanding of multiple languages.

**Figure 4:**
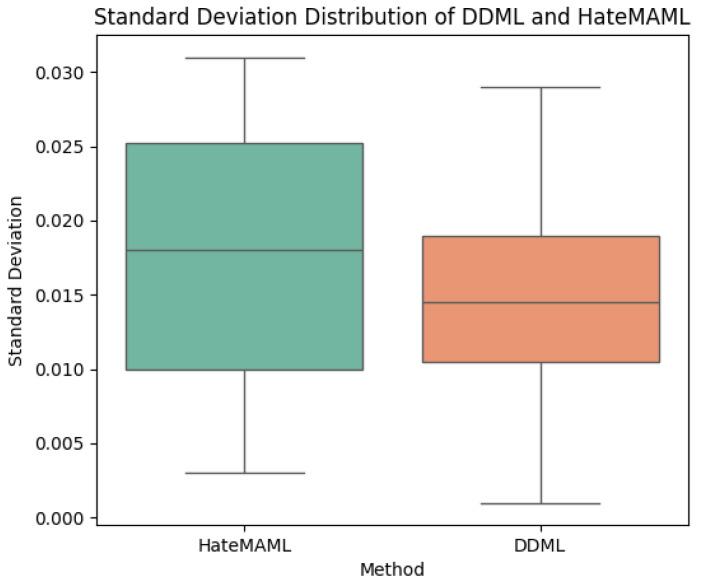
Standard deviation distributions of DDML and HateMAML.

From the perspective of standard deviation, deep neural networks trained with DDML generally exhibit lower standard deviations, indicating that DDML has stronger stability and less fluctuation. Compared to HateMAML, DDML demonstrates better stability, as the F1 score and accuracy of HateMAML both tend to fluctuate more, suggesting that it may be more sensitive to the training data or hyperparameters. The box plots of the standard deviation distributions for both training methods is shown in Figure 4.

### 5.2. Comparison Experiment Between DDML and CLHSD

In order to replicate the CLHSD experiment by Firmino et al. [42] and compare it with DDML, this experiment adopted the same methodology as the original paper, using the Haspeede dataset as the source language and the OffComBr-2 dataset as the target language. Additionally, we replicated the four experimental strategies used in the original paper: joint learning (JL), cascaded learning (CL), combined CL/JL (combining 70% of the source dataset with 30% of the target dataset), and CL/JL+ (initial training cycle using the same data split as CL/JL, followed by subsequent training cycles in which the remaining language corpus is split based on the number of training cycles using k-fold cross-validation).

Joint learning is a machine learning paradigm in which the core idea is to simultaneously optimize multiple related tasks or objectives within the same training process, thereby enabling models to leverage information from different tasks to enhance overall performance. Joint learning has widespread applications in multitask learning, cross-domain learning, knowledge distillation, and other related fields. Cascaded learning is another machine learning paradigm; in CL, the core idea is to decompose the learning task into multiple stages, with the model for each stage relying on the output of the previous stage to gradually optimize the final prediction result. This method can improve the efficiency and accuracy of the model while reducing the computational complexity. It is widely used in computer vision, natural language processing, reinforcement learning, and other fields.

**Figure 5:**
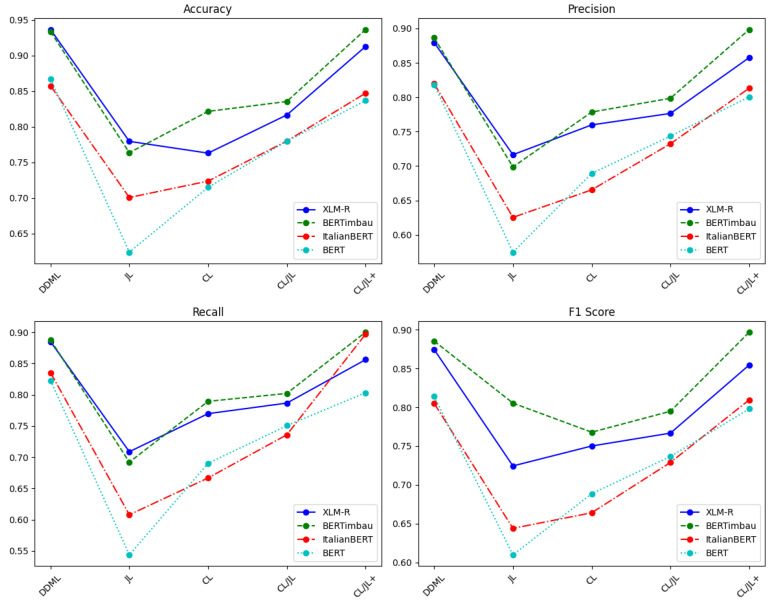
Comparison of experimental results between DDML and CLHSD on the OffComBr-2 dataset.

The comparison between DDML and CLHSD on the OffComBr-2 dataset is shown in Table 5 and Table 6 and Figure 5. From the table data, it is evident that DDML performs significantly better than CLHSD with each of the JL, CL, CL/JL, and CL/JL+ strategies. On four different deep neural networks (XLM-R, BERTimbau, ItalianBERT, and BERT), the DDML training method exhibits the best performance in both accuracy and F1 score or at least matches the CL/JL+ method. On all models, the F1 score under DDML is either the highest or comparable to the CL/JL+ method, indicating that DDML not only improves classification accuracy but also ensures the robustness of the model, allowing it to maintain strong performance across different test sets. By observing the precision and recall results, it can be seen that the network trained with DDML achieves balanced performance between the two metrics, with both results being close to the F1 score. This indicates that the DDML model does not favor optimizing precision at the cost of recall, nor does it prioritize recall at the expense of precision.; in other words, the model reaches a good compromise between the two metrics, resulting in a relatively ideal outcome.

In conclusion, DDML outperforms CLHSD on the OffComBr-2 dataset. DDML shows stronger advantages in both accuracy and F1 score, effectively enhancing the performance of hate speech detection tasks while also demonstrating greater generalization ability and stability.

### 5.3. Comparison Experiment Between DDML and TLHSD

In their research based on TLHSD, Fillies et al. [43] used GermanBERT, ItalianBERT, and BETO on the GermEval 2018, Haspeede, and HaterNET datasets. For the experiments reported in this subsection, we used the corresponding networks on the same three datasets. Additionally, as a comparison, we used XLM-R (xlm-roberta-base) as the student network. For comparison with the study by Fillies et al. [43], our student network used distillBERT on the Multilingual, GoogleMultilingual, and DeeplMultilingual datasets, as was the case in their research.

**Figure 6:**
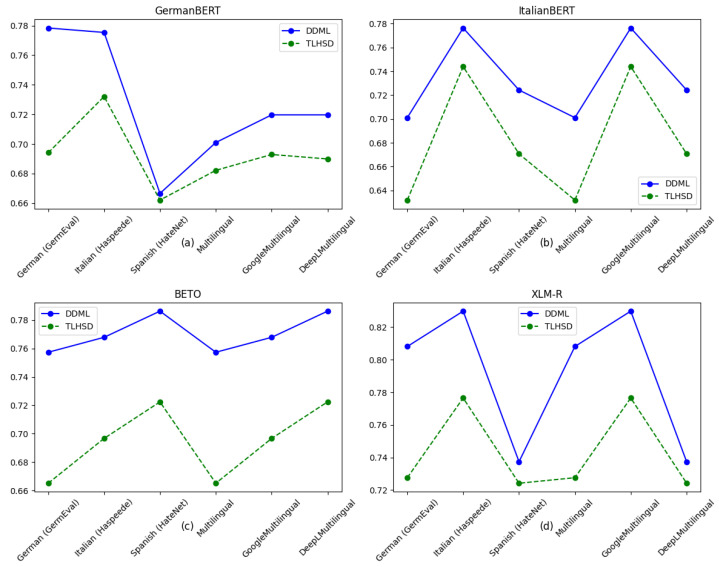
Comparison of F1 score results between DDML and TLHSD on the GermEval, Haspeede, and HaterNet datasets. In this figure, subfigures (**a**–**d**) represent the F1 Score performance of GermanBERT, ItalianBERT, BETO, and XLM-R on six datasets: German (GermEval), Italian (Haspeede), Spanish (HaterNet), Multilingual, GoogleMultilingual, and DeepLMultilingual, respectively. The blue line represents the F1 Score of the model trained with DDML, while the green line represents the F1 Score of the model trained with TLHSD.

The results of our experiment comparing DDML and TLHSD are shown in Table 7 and Table 8 and in Figure 6. From the results in Table 7, it can be seen that DDML outperforms TLHSD on all three datasets. In terms of accuracy, DDML demonstrates higher accuracy on the three different language datasets, indicating its better ability to adapt to different language environments and improve the model’s overall classification ability. Regarding the F1 score, DDML achieves a higher F1 score on all datasets compared to TLHSD, demonstrating that the neural networks trained with DDML exhibit more balanced performance across different datasets.

The experimental results in Table 8 show that DDML outperforms TLHSD across all datasets and evaluation metrics in terms of both accuracy and F1 score. DDML performs exceptionally well with the XLM-R model, achieving an accuracy of over 0.75 on multiple datasets, which is significantly higher than the results for TLHSD. This indicates that DDML can effectively learn cross-lingual hate speech detection features on large and complex datasets, resulting in improved detection performance.

In summary, the experimental results of DDML on all monolingual and multilingual datasets demonstrate that it outperforms TLHSD in both accuracy and F1 score. Notably, DDML shows significant improvement on the Italian and Spanish datasets, suggesting that our proposed method exhibits better feature learning capabilities, stronger generalization, and greater stability in multilingual hate speech detection tasks.

### 5.4. Experiments Applying DDML to Racism and Sexism Datasets

**Figure 7:**
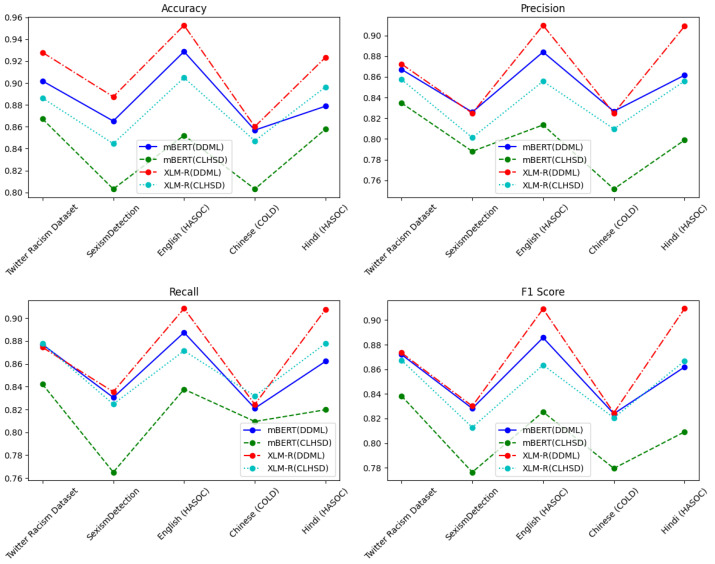
Comparison of experimental results between DDML and TLHSD (CL/JL+) on the Twitter Racism, SexismDetection, English (HASOC), Chinese (COLD), and Hindi (HASOC) datasets.

The experiments described in this subsection tested the performance of DDML on different racism and sexism datasets. Because Awal et al. [41] did not use the Hindi (HASOC) and English (HASOC) datasets in their study on HateMAML, and as the previous sections did not include any experiments on Chinese-language datasets, we included the Hindi (HASOC) and Chinese (COLD) datasets for the experiments in this section. To validate the effectiveness of DDML, we used CLHSD (CL/JL+), which performed the best in the previous experiments, as a comparison, and trained the model using Italian (Haspeede) as the source language. The experimental results are shown in Table 9 and Figure 7.

DDML achieved better results across the different datasets, particularly in terms of the key F1 score evaluation metric, where it consistently outperformed CLHSD (CL/JL+) in all experiments. This indicates that our proposed training approach can effectively enhance the overall predictive capability of the resulting model.

In classification tasks, there is usually a tradeoff between precision and recall, with methods tending to either improve precision at the cost of recall or vice versa. Our proposed DDML achieves a good balance between precision and recall, allowing the model to achieve improved prediction accuracy without significantly sacrificing recall.

Across different models, DDML not only performs well on mBERT but also demonstrates stable performance improvements on XLM-R, a more powerful multilingual model.

Due to the limited availability of training data, it is generally more challenging to train models for low-resource languages such as Hindi and Chinese in comparison to high-resource languages such as English. This makes it difficult for the resulting models to learn sufficient semantic and syntactic patterns. Despite this, DDML still achieves strong performance on the Hindi dataset.

In summary, DDML outperforms CLHSD (CL/JL+) across multiple dimensions, including overall performance, precision–recall balance, generalization capability, and adaptability to low-resource languages, demonstrating its superior training strategy.

### 5.5. DDML Error Analysis

Models from both the BERT series and the XLM-R series outperform the baseline when trained with DDML. Performance is improved in terms of both overall accuracy and F1 score, while achieving a better balance between precision and recall. However, the mBERT model trained with DDML is insufficiently competitive on two datasets, namely, the Greek and Turkish HASOC datasets.

Our analysis indicates that the main reasons for this are as follows:

1. Insufficient coverage of pretraining data for the language model: mBERT may not have adequately covered the specific domains or corpora of the Greek and Turkish languages during pretraining. This could have resulted in mBERT acquiring a weaker understanding of these two languages, affecting the performance of DDML on these datasets.

2. Imbalanced sample distribution in the datasets: In the case of imbalanced samples in these two datasets, the model may have predicted the more frequent category (i.e., negative samples), leading to weaker prediction performance for positive samples. This can be corroborated by the lower recall results.

## 6. Conclusions

This paper is the first to propose the use of knowledge distillation for offensive language identification, introducing a novel distillation framework named DDML. We conducted classification experiments using data from nine datasets and six pretrained models from HuggingFace spanning a total of ten languages. The results show that our proposed DDML significantly outperforms baseline methods. Our experiments also reveal that models trained with DDML consistently achieve better average performance with XLM-R compared to BERT-based networks across both monolingual and multilingual datasets. Thus, XLM-R models should be prioritized when developing hate speech detectors. Moreover, we believe that DDML has broader applicability beyond hate speech detection, and could be used effectively in any domain where network-based problem solving is required.

However, the proposed DDML still has several aspects worth discussing and exploring further in future work:

1. Model efficiency tradeoffs: DDML uses a powerful teacher network and two or more smaller student networks. Even though the teacher network does not participate in backpropagation or parameter updates during training, the process still requires a significant amount of computational resources. Therefore, future work could introduce tradeoffs between training complexity and model performance with the aim of reducing the computational resources required for training while maintaining model performance as much as possible. We believe that there are two approaches worth considering in this regard. The first is to reduce the number of parameters in the student network by using RNN and LSTM networks, which also have strong sequence modeling capabilities. This can reduce the student network’s parameter size from 100 M–200 M to below 10 M. The second approach involves reducing the number of parameters in the teacher network. Similar to XLM-RoBERTa-Large, which we used in this paper, Google’s T5 series models [46] are large-scale pretrained language models; however, T5 models have only around 200 M parameters, which would significantly reduce the computational burden compared to the 561 M parameters of xlm-roBERTa-large.

2. The performance of mBERT on the Turkish and Greek datasets is insufficiently competitive: The mBERT model trained with DDML did not perform competitively on Turkish and Greek datasets. We believe that future research could address this issue by applying data augmentation to these two datasets and conducting more pretraining of the student networks for these languages.

## Figures and Tables

**Table 1:** Statistics for the monolingual datasets.

Dataset	Offensive	Non-Offensive	Total
Arabic (SemEval)	1919	7747	9666
Danish (SemEval)	425	2864	3289
Greek (SemEval)	1401	3378	4779
Turkish (SemEval)	6757	28,035	34,792
English (HASOC)	2243	2279	4522
German (HASOC)	807	2092	2899
Hindi (HASOC)	1044	2582	3626
English (OLID)	4640	9460	14,100
Chinese (COLD)	14,830	16,219	31,049
German (GermEval)	2890	5651	8541
Italian (HaSpeede)	3472	5126	8598
Spanish (HaterNet)	1567	4433	6000
Portuguese (OffComBR-2)	419	831	1250
English (Twitter Racisim Dataset)	11,501	7747	19,248
English (SexismDetection)	4340	13,498	17,838

**Table 2:** Statistics for the multilingual datasets.

Dataset	Offensive	Non-Offensive	Total
UniHate	21,504	62,279	84,783
Multilingual	7929	15,210	23,139
GoogleMultilingual	7929	15,210	23,139
DeeplMultilingual	7929	152,10	23,139

**Table 3:** Transformer-based model parameters.

Model	Parameters
XLM-R (xlm-roberta-large)	561 M
XLM-R (xlm-roberta-base)	279 M
mBERT (bert-base-multilingual-uncased)	168 M
distillBERT (distilbert-base-uncased)	67 M
GermanBERT (bert-base-german-cased)	110 M
ItalianBERT (bert-base-italian-cased)	111 M
BETO (bert-base-spanish-wwm-cased)	67 M
BERTimbau (bert-base-portuguese-cased)	110 M

**Table 4:** Comparison of experimental results for DDML and HateMAML on the OLID, SemEval2020, HaSpeedDe, and HASOC2020 datasets. In this table, the bolded parts are the well-performing ones in this set of experiments, results marked with ▴ represent the DDML results and those marked with • represent the HateMAML results.

Dataset	mBERT				XLM-R			
	Accuracy	Precision	Recall	F1 Score	Accuracy	Precision	Recall	F1 Score
▴ English (OLID)	$0.8213_{0.031}$	$0.7860_{0.015}$	$0.8167_{0.026}$	$0.8009_{0.032}$	$0.8735_{0.002}$	$0.7964_{0.002}$	$0.8016_{0.005}$	$0.8011_{0.007}$
• English (OLID)	$0.6875_{0.012}$	$0.6962_{0.014}$	$0.6767_{0.021}$	$0.6863_{0.012}$	$0.7274_{0.019}$	$0.7099_{0.011}$	$0.7353_{0.025}$	$0.7224_{0.028}$
▴ Arabic (SemEval)	$0.8469_{0.026}$	$0.8276_{0.002}$	$0.8206_{0.027}$	$0.8241_{0.029}$	$0.8671_{0.012}$	$0.8259_{0.005}$	$0.8239_{0.016}$	$0.8249_{0.013}$
• Arabic (SemEval)	$0.7531_{0.014}$	$0.7509_{0.024}$	$0.7880_{0.031}$	$0.7690_{0.022}$	$0.5142_{0.005}$	$0.7398_{0.033}$	$0.3759_{0.030}$	$0.4985_{0.010}$
▴ Danish (SemEval)	$0.7856_{0.016}$	$0.7524_{0.017}$	$0.7710_{0.018}$	$0.7616_{0.024}$	$0.8162_{0.006}$	$0.7705_{0.012}$	$0.7737_{0.010}$	$0.7721_{0.008}$
• Danish (SemEval)	$0.7091_{0.018}$	$0.7277_{0.012}$	$0.6831_{0.013}$	$0.7047_{0.025}$	$0.7366_{0.021}$	$0.7625_{0.029}$	$0.7085_{0.033}$	$0.7345_{0.011}$
▴ Turkish (SemEval)	$0.5734_{0.025}$	$0.6496_{0.035}$	$0.3369_{0.026}$	$0.4437_{0.027}$	$0.8019_{0.020}$	$0.7652_{0.023}$	$0.8056_{0.018}$	$0.7849_{0.019}$
• Turkish (SemEval)	$0.5465_{0.008}$	$0.7074_{0.019}$	$0.4061_{0.011}$	$0.5160_{0.003}$	$0.6261_{0.022}$	$0.7498_{0.020}$	$0.4991_{0.024}$	$0.5993_{0.026}$
▴ Greek (SemEval)	$0.9093_{0.018}$	$0.6867_{0.017}$	$0.3854_{0.012}$	$0.4937_{0.031}$	$0.9094_{0.019}$	$0.7214_{0.021}$	$0.3937_{0.021}$	$0.5094_{0.013}$
• Greek (SemEval)	$0.6564_{0.010}$	$0.6467_{0.020}$	$0.6252_{0.026}$	$0.6358_{0.014}$	$0.6536_{0.006}$	$0.6072_{0.021}$	$0.6248_{0.023}$	$0.6159_{0.009}$
▴ German (HASOC)	$0.8565_{0.001}$	$0.8082_{0.023}$	$0.8168_{0.007}$	$0.8125_{0.011}$	$0.8762_{0.009}$	$0.8567_{0.026}$	$0.8390_{0.005}$	$0.8478_{0.016}$
• German (HASOC)	$0.7962_{0.027}$	$0.7879_{0.020}$	$0.7668_{0.016}$	$0.7772_{0.029}$	$0.8241_{0.006}$	$0.7929_{0.021}$	$0.7893_{0.018}$	$0.7911_{0.023}$
▴ Italian-news (Haspeede)	$0.7623_{0.014}$	$0.7432_{0.022}$	$0.7293_{0.008}$	$0.7362_{0.005}$	$0.7953_{0.014}$	$0.7555_{0.016}$	$0.7509_{0.018}$	$0.7532_{0.008}$
• Italian-news (Haspeede)	$0.7051_{0.030}$	$0.7991_{0.029}$	$0.6283_{0.030}$	$0.6427_{0.026}$	$0.7124_{0.016}$	$0.6926_{0.008}$	$0.6709_{0.009}$	$0.6816_{0.010}$
▴ Italian-tweets (Haspeede)	$0.7620_{0.015}$	$0.7699_{0.014}$	$0.7691_{0.021}$	$0.7695_{0.019}$	$0.8025_{0.027}$	$0.7829_{0.010}$	$0.7702_{0.011}$	$0.7765_{0.012}$
• Italian-tweets (Haspeede)	$0.5953_{0.031}$	$0.6338_{0.024}$	$0.5054_{0.022}$	$0.5624_{0.018}$	$0.7354_{0.021}$	$0.6959_{0.030}$	$0.6654_{0.018}$	$0.6803_{0.027}$
▴ UniHate	$0.8044_{0.016}$	$0.7799_{0.020}$	$0.7873_{0.011}$	$0.7836_{0.017}$	$0.8452_{0.017}$	$0.8053_{0.023}$	$0.8101_{0.018}$	$0.8068_{0.022}$
• UniHate	$0.7365_{0.022}$	$0.6887_{0.021}$	$0.7119_{0.015}$	$0.7001_{0.025}$	$0.7996_{0.029}$	$0.7516_{0.034}$	$0.7663_{0.023}$	$0.7589_{0.018}$

**Table 5:** Comparison of experimental results (accuracy and F1 score) between DDML and CLHSD on the OffComBr-2 dataset. In this table, the bolded parts are the well-performing ones in this set of experiments.

Training Method	XLM-R		BERTimbau		ItalianBERT		BERT	
	Accuracy	F1 Score	Accuracy	F1 Score	Accuracy	F1 Score	Accuracy	F1 Score
**DDML**	$0.9364_{0.025}$	$0.8795_{0.002}$	$0.9334_{0.014}$	$0.8864_{0.028}$	$0.8573_{0.019}$	$0.8198_{0.024}$	$0.8867_{0.012}$	$0.8183_{0.007}$
**CLHSD(JL)**	$0.7794_{0.010}$	$0.7165_{0.004}$	$0.7635_{0.015}$	$0.6987_{0.004}$	$0.7006_{0.020}$	$0.6254_{0.008}$	$0.6238_{0.011}$	$0.5748_{0.029}$
**CLHSD(CL)**	$0.7629_{0.025}$	$0.7598_{0.017}$	$0.8215_{0.026}$	$0.7785_{0.029}$	$0.7236_{0.029}$	$0.6654_{0.017}$	$0.7149_{0.005}$	$0.6894_{0.027}$
**CLHSD(CL/JL)**	$0.8165_{0.020}$	$0.7765_{0.027}$	$0.8354_{0.026}$	$0.7985_{0.021}$	$0.7798_{0.026}$	$0.7324_{0.007}$	$0.7796_{0.011}$	$0.7435_{0.014}$
**CLHSD(CL/JL+)**	$0.9127_{0.030}$	$0.8576_{0.026}$	$0.9365_{0.017}$	$0.8985_{0.026}$	$0.8471_{0.019}$	$0.8132_{0.029}$	$0.8366_{0.004}$	$0.8006_{0.021}$

**Table 6:** Comparison of experimental results (precision and recall) between DDML and CLHSD on the OffComBr-2 dataset. In this table, the bolded parts are the well-performing ones in this set of experiments.

Training Method	XLM-R		BERTimbau		ItalianBERT		BERT	
	Precision	Recall	Precision	Recall	Precision	Recall	Precision	Recall
**DDML**	$0.8847_{0.013}$	$0.8744_{0.012}$	$0.8874_{0.020}$	$0.8854_{0.003}$	$0.8352_{0.011}$	$0.8050_{0.017}$	$0.8227_{0.016}$	$0.8139_{0.011}$
**CLHSD(JL)**	$0.7088_{0.020}$	$0.7244_{0.027}$	$0.6921_{0.023}$	$0.7054_{0.013}$	$0.6079_{0.026}$	$0.6439_{0.026}$	$0.5437_{0.025}$	$0.6097_{0.031}$
**CLHSD(CL)**	$0.7697_{0.022}$	$0.7501_{0.033}$	$0.7894_{0.028}$	$0.7679_{0.029}$	$0.6668_{0.018}$	$0.6640_{0.022}$	$0.6902_{0.029}$	$0.6886_{0.018}$
**CLHSD(CL/JL)**	$0.7866_{0.031}$	$0.7667_{0.016}$	$0.8021_{0.017}$	$0.7949_{0.002}$	$0.7359_{0.009}$	$0.7289_{0.025}$	$0.7505_{0.020}$	$0.7366_{0.032}$
**CLHSD(CL/JL+)**	$0.8561_{0.008}$	$0.8546_{0.009}$	$0.9001_{0.029}$	$0.8969_{0.023}$	$0.8168_{0.020}$	$0.8096_{0.018}$	$0.8032_{0.001}$	$0.7980_{0.007}$

**Table 7:** Experimental results comparing DDML and TLHSD on the GermEval, Haspeede, and HaterNet datasets. In this table, the bolded parts are the well-performing ones in this set of experiments.

Dataset	Method	Metrics	GermanBERT	ItalianBERT	BETO	XLM-R
**German** **(GermEval)**	DDML	Accuracy	$0.7796_{0.022}$	$0.7542_{0.005}$	$0.7885_{0.010}$	$0.8356_{0.021}$
		Precision	$0.7840_{0.007}$	$0.6978_{0.012}$	$0.7561_{0.011}$	$0.8064_{0.020}$
		Recall	$0.7729_{0.006}$	$0.7044_{0.018}$	$0.7585_{0.003}$	$0.8098_{0.016}$
		F1 Score	$0.7784_{0.020}$	$0.7011_{0.002}$	$0.7573_{0.003}$	$0.8081_{0.012}$
	TLHSD	Accuracy	$0.7264_{0.028}$	$0.7089_{0.017}$	$0.6988_{0.022}$	$0.7885_{0.015}$
		Precision	$0.6820_{0.033}$	$0.6188_{0.014}$	$0.6643_{0.029}$	$0.7236_{0.025}$
		Recall	$0.7066_{0.012}$	$0.6456_{0.005}$	$0.6661_{0.016}$	$0.7316_{0.020}$
		F1 Score	$0.6941_{0.021}$	$0.6319_{0.022}$	$0.6652_{0.012}$	$0.7276_{0.008}$
**Italian** **(Haspeede)**	DDML	Accuracy	$0.7773_{0.009}$	$0.7933_{0.019}$	$0.7678_{0.019}$	$0.8301_{0.029}$
		Precision	$0.7718_{0.003}$	$0.7731_{0.004}$	$0.7651_{0.019}$	$0.8293_{0.017}$
		Recall	$0.7790_{0.014}$	$0.7797_{0.010}$	$0.7705_{0.020}$	$0.8303_{0.013}$
		F1 Score	$0.7754_{0.015}$	$0.7764_{0.017}$	$0.7678_{0.024}$	$0.8298_{0.021}$
	TLHSD	Accuracy	$0.7356_{0.009}$	$0.7462_{0.021}$	$0.7052_{0.025}$	$0.7884_{0.021}$
		Precision	$0.7159_{0.023}$	$0.7367_{0.013}$	$0.6902_{0.009}$	$0.7733_{0.010}$
		Recall	$0.7490_{0.025}$	$0.7514_{0.011}$	$0.7033_{0.019}$	$0.7797_{0.028}$
		F1 Score	$0.7321_{0.006}$	$0.7440_{0.023}$	$0.6967_{0.011}$	$0.7765_{0.027}$
**Spanish** **(HaterNet)**	DDML	Accuracy	$0.7517_{0.022}$	$0.7617_{0.010}$	$0.7881_{0.026}$	$0.7654_{0.009}$
		Precision	$0.6688_{0.018}$	$0.7296_{0.006}$	$0.7844_{0.010}$	$0.7426_{0.016}$
		Recall	$0.6644_{0.017}$	$0.7191_{0.005}$	$0.7853_{0.008}$	$0.7323_{0.018}$
		F1 Score	$0.6666_{0.016}$	$0.7243_{0.004}$	$0.7862_{0.030}$	$0.7374_{0.008}$
	TLHSD	Accuracy	$0.7026_{0.019}$	$0.7281_{0.004}$	$0.7263_{0.016}$	$0.7633_{0.011}$
		Precision	$0.6095_{0.010}$	$0.6646_{0.022}$	$0.7105_{0.010}$	$0.7123_{0.022}$
		Recall	$0.6352_{0.025}$	$0.6769_{0.010}$	$0.7347_{0.022}$	$0.7364_{0.015}$
		F1 Score	$0.6221_{0.026}$	$0.6707_{0.011}$	$0.7224_{0.008}$	$0.7242_{0.019}$

**Table 8:** Comparison of experimental results between DDML and TLHSD on the Multilingual, GoogleMultilingual, and DeepLMultilingual datasets. In this table, the bolded parts are the well-performing ones in this set of experiments.

Dataset	Method	Metrics	mBERT	distillBERT	XLM-R
**Multilingual**	DDML	Accuracy	$0.7612_{0.007}$	$0.6993_{0.009}$	$0.7561_{0.002}$
		Precision	$0.6999_{0.018}$	$0.6770_{0.011}$	$0.7495_{0.006}$
		Recall	$0.7021_{0.017}$	$0.6788_{0.002}$	$0.7501_{0.002}$
		F1 Score	$0.7010_{0.023}$	$0.6779_{0.005}$	$0.7498_{0.005}$
	TLHSD	Accuracy	$0.6965_{0.024}$	$0.6960_{0.018}$	$0.7321_{0.020}$
		Precision	$0.6735_{0.028}$	$0.6541_{0.015}$	$0.6910_{0.019}$
		Recall	$0.6911_{0.016}$	$0.6611_{0.028}$	$0.7154_{0.012}$
		F1 Score	$0.6822_{0.023}$	$0.6576_{0.025}$	$0.7030_{0.018}$
**GoogleMultilingual**	DDML	Accuracy	$0.7636_{0.026}$	$0.7533_{0.019}$	$0.8087_{0.027}$
		Precision	$0.7232_{0.019}$	$0.7572_{0.010}$	$0.7609_{0.014}$
		Recall	$0.7162_{0.016}$	$0.7556_{0.021}$	$0.7633_{0.011}$
		F1 Score	$0.7197_{0.018}$	$0.7564_{0.017}$	$0.7621_{0.007}$
	TLHSD	Accuracy	$0.7414_{0.012}$	$0.7281_{0.010}$	$0.7662_{0.012}$
		Precision	$0.6804_{0.026}$	$0.7088_{0.011}$	$0.7281_{0.008}$
		Recall	$0.7059_{0.005}$	$0.7289_{0.021}$	$0.7398_{0.011}$
		F1 Score	$0.6929_{0.006}$	$0.7187_{0.016}$	$0.7339_{0.005}$
**DeepLMultilingual**	DDML	Accuracy	$0.7636_{0.026}$	$0.7533_{0.019}$	$0.8087_{0.027}$
		Precision	$0.7310_{0.011}$	$0.7581_{0.013}$	$0.7926_{0.010}$
		Recall	$0.7087_{0.016}$	$0.7547_{0.018}$	$0.7596_{0.004}$
		F1 Score	$0.7197_{0.018}$	$0.7564_{0.017}$	$0.7621_{0.007}$
	TLHSD	Accuracy	$0.7011_{0.027}$	$0.7124_{0.016}$	$0.7481_{0.010}$
		Precision	$0.6598_{0.004}$	$0.7013_{0.025}$	$0.7226_{0.019}$
		Recall	$0.6745_{0.012}$	$0.6798_{0.011}$	$0.7009_{0.018}$
		F1 Score	$0.6899_{0.015}$	$0.6904_{0.014}$	$0.7116_{0.018}$

**Table 9:** Comparison of experimental results between DDML and CLHSD (CL/JL+) on the Twitter Racism, SexismDetection, English (HASOC), Chinese (COLD), and Hindi (HASOC) datasets. In this table, the bolded parts are the well-performing ones in this set of experiments, results marked with ▴ represent the DDML results, while results marked with • represent the CLHSD (CL/JL+) results.

Dataset	mBERT				XLM-R			
	Accuracy	Precision	Recall	F1 Score	Accuracy	Precision	Recall	F1 Score
▴ Twitter Racism Dataset	$0.9018_{0.007}$	$0.8672_{0.011}$	$0.8769_{0.016}$	$0.8720_{0.014}$	$0.9279_{0.002}$	$0.8724_{0.008}$	$0.8744_{0.009}$	$0.8734_{0.010}$
• Twitter Racism Dataset	$0.8671_{0.023}$	$0.8347_{0.021}$	$0.8421_{0.019}$	$0.8384_{0.031}$	$0.8862_{0.028}$	$0.8574_{0.024}$	$0.8776_{0.022}$	$0.8674_{0.021}$
▴ SexismDetection	$0.8652_{0.031}$	$0.8259_{0.025}$	$0.8305_{0.030}$	$0.8282_{0.031}$	$0.8874_{0.015}$	$0.8248_{0.017}$	$0.8355_{0.009}$	$0.8301_{0.015}$
• SexismDetection	$0.8031_{0.025}$	$0.7880_{0.031}$	$0.7651_{0.024}$	$0.7764_{0.027}$	$0.8445_{0.019}$	$0.8012_{0.022}$	$0.8247_{0.027}$	$0.8128_{0.020}$
▴ English (HASOC)	$0.9287_{0.011}$	$0.8840_{0.030}$	$0.8874_{0.027}$	$0.8857_{0.030}$	$0.9527_{0.003}$	$0.9098_{0.022}$	$0.9084_{0.020}$	$0.9091_{0.023}$
• English (HASOC)	$0.8521_{0.021}$	$0.8135_{0.021}$	$0.8377_{0.019}$	$0.8254_{0.025}$	$0.9048_{0.030}$	$0.8557_{0.024}$	$0.8714_{0.018}$	$0.8635_{0.028}$
▴ Chinese (COLD)	$0.8569_{0.009}$	$0.8267_{0.020}$	$0.8211_{0.022}$	$0.8239_{0.016}$	$0.8601_{0.013}$	$0.8248_{0.019}$	$0.8244_{0.028}$	$0.8246_{0.027}$
• Chinese (COLD)	$0.8031_{0.024}$	$0.7516_{0.025}$	$0.8095_{0.021}$	$0.7795_{0.021}$	$0.8471_{0.018}$	$0.8097_{0.031}$	$0.8318_{0.025}$	$0.8206_{0.017}$
▴ Hindi (HASOC)	$0.8788_{0.009}$	$0.8616_{0.009}$	$0.8622_{0.006}$	$0.8619_{0.012}$	$0.9235_{0.013}$	$0.9090_{0.015}$	$0.9077_{0.009}$	$0.9064_{0.011}$
• Hindi (HASOC)	$0.8579_{0.021}$	$0.7989_{0.015}$	$0.8198_{0.019}$	$0.8092_{0.025}$	$0.8964_{0.027}$	$0.8560_{0.027}$	$0.8779_{0.018}$	$0.8668_{0.020}$

## Data Availability

The data presented in this study are available on request from the corresponding author.
